# High-Performance Indigenous *Lactiplantibacillus plantarum* Strains for Enhanced Malolactic Fermentation and Wine Quality

**DOI:** 10.3390/microorganisms13102328

**Published:** 2025-10-09

**Authors:** Yongzhang Zhu, Ni Chen, Zhenghua Xu, Jingyue Liu, Shuwen Liu, Kan Shi

**Affiliations:** 1 Guangdong Provincial Key Laboratory of Intelligent Port Security Inspection, Huangpu Customs District P.R. China, Guangzhou 510700, China; zhuyongzhang@nwafu.edu.cn (Y.Z.); xuzhciq@163.com (Z.X.); 2 College of Enology, Shaanxi Engineering Research Center for Viti-Viniculture, Viti-Viniculture Engineering Technology Center of State Forestry and Grassland Administration, Heyang Experimental and Demonstrational Stations for Grape, Ningxia Helan Mountain’s East Foothill Wine Experiment and Demonstration Station, Northwest A&F University, Yangling 712100, China; liujingyue2025@163.com; 3 Shaanxi Modern Agriculture Training Center, Xi’an 710000, China; chenni1004@163.com

**Keywords:** MLF, lactic acid bacteria, *L. plantarum*, stress tolerance, aromas

## Abstract

Malolactic fermentation (MLF), a key enological process for wine deacidification and aroma and flavor development, is predominantly mediated by lactic acid bacteria. This study characterized 342 indigenous *Lactiplantibacillus plantarum* (*L. plantarum*) isolates, a potential starter species underexploited for MLF, from China’s Jiaodong Peninsula wine regions through polyphasic analysis. Thirty strains with high tolerance to wine stress conditions and efficient malate metabolism were selected. Among these, two high-performance strains, P101 and J43, exhibited superior MLF kinetics. Their applications had almost no effect on the wine’s basic physicochemical parameters, color parameters, and individual phenolic contents. Solid-phase microextraction–gas chromatography–mass spectrometry (SPME-GC-MS) analysis revealed that these strains significantly enhance key aroma compound contents in wines, including ethyl acetate, ethyl lactate, ethyl 2-methylbutyrate, and nerol, contributing more floral and fruity aroma characteristics. These indigenous *L. plantarum* strains, novel microbial starter cultures, demonstrate dual functionality in enhancing wine quality through controlled fermentation while supporting microbial biodiversity through the development of region-specific strain resources.

## 1. Introduction

Malolactic fermentation (MLF) is a critical process in winemaking, an enzymatic decarboxylation of L-malic acid to L-lactic acid and carbon dioxide (CO_2_), contributing to acidity reduction, flavor enhancement, increased complexity, and improved microbial stability [1,2]. This transformation is crucial for the production of the majority of red wines and selected white wines [3].

During winemaking, the primary lactic acid bacteria (LAB) genera frequently encountered include *Oenococcus* spp., *Lactobacillus* spp., *Pediococcus* spp., and *Leuconostoc* spp. [4,5]. Among these, *Oenococcus oeni* (*O. oeni*) dominates due to its superior adaptability to the wine environment [6,7]. *Lactiplantibacillus plantarum* (*L. plantarum*) exhibits a certain ability to survive in the harsh wine environment [8,9]. Among its notable traits, the ability to produce bacteriocins stands as a key advantage [10]. This trait can aid in maintaining strain dominance during fermentation and reducing reliance on SO_2_ [11]. Furthermore, *L. plantarum* exhibits a more diverse repertoire of wine-associated enzymes compared to *O. oeni* [12], a feature that enhances wine aroma and subsequently improves overall sensory quality [13]. However, *L. plantarum* remains underutilized as a wine fermentation starter, with only the ML Prime™ strain from LALLEMAND finding commercial application in fermentation agents. This limited adoption stems from its susceptibility to low pH and high ethanol levels. Rising alcohol levels in wines, driven by global warming, coupled with the cool climatic conditions prevalent in northern China, present a growing challenge to the survival and fermentation efficiency of malolactic bacteria (MLB) [14,15]. Additionally, the prevalent utilization of commercial starter cultures across global wine regions may diminish regional distinctiveness and increase product homogeneity. In recent years, major wine-producing countries such as Spain, Italy, Argentina, and Chile have increasingly prioritized the development and application of indigenous MLB strains [16,17,18,19,20,21,22,23]. Thus, identifying novel potential *L. plantarum* strains capable of adapting to winemaking stressors holds significant importance for China’s wine industry. The Jiaodong Peninsula—encompassing key regions such as Qingdao and Yantai—ranks among China’s premier grape-growing areas, boasting exceptionally favorable geographical and climatic conditions [24,25]. However, the indigenous MLB resources of this region have not yet been systematically explored.

Herein, we performed a comprehensive analysis and screening of 342 *L. plantarum* strains isolated from the Jiaodong Peninsula under stress conditions. Two superior strains, J43 and P101, were subjected to a thorough evaluation of their fermentation performance and their impacts on multiple aspects of Marselan wine. Our research contributes to the discovery of novel, elite indigenous LAB strains in China and enhances our understanding of their MLF characteristics, thereby providing technical and experimental foundations for the innovation of wine LAB starter cultures and the improvement of wine region characteristics and quality.

## 2. Materials and Methods

### 2.1. Experimental Strains and Culture Conditions

A total of 342 *L. plantarum* strains were isolated from spontaneous MLF wine samples of different grape varieties collected from wineries in the Jiaodong Peninsula, specifically including the following: Chateau Nine Peaks (Chardonnay; 54 strains); Pula Valley (Chardonnay and Marselan; 52 strains); Taiyihu Winery (Chardonnay, Cabernet Sauvignon, and Marselan; 104 strains); Greatwall Longji Winery (Chardonnay and Marselan; 31 strains); and Aweihai Winery (Marselan; 91 strains). *L. plantarum* XJ25, preserved in the Microbiology Laboratory of the College of Enology at Northwest A&F University, was used as the type strain [26]. For the identification and classification of the strains, we employed a specific PCR. The primers used were as follows: upstream primer, 5′-TTGCCACCAACCATTCAGC-3′, and downstream primer, 5′-CTAACCATGATGATAATCGA-3′ [27]. Furthermore, an MRS medium was employed for the isolation of strains. All *L. plantarum* strains were cultured in an MRS medium under optimal conditions: 37 °C in a constant-temperature anaerobic incubator for 24 h. The MRS liquid medium composition was as follows: peptone (10 g/L), beef extract (10 g/L), glucose (20 g/L), yeast extract (5 g/L), CH_2_COONa (5 g/L), K_2_HPO_4_ (2 g/L), diammonium hydrogen citrate (2 g/L), MgSO_4_·7H_2_O (0.2 g/L), MnSO_4_·H_2_O (0.05 g/L), and Tween 80 (1 mL/L). The medium was sterilized at 115 °C for 15 min prior to use. For the MRS solid medium, 15 g of agar was added to 1 L of the liquid medium.

### 2.2. Stress Tolerance Analysis of L. plantarum Strains

The simulated wine contained 10 mL/L grape juice, 2 g/L glucose, 2 g/L D-fructose, 0.2 g/L NaCl, 1 g/L (NH_4_)_2_SO_4_, 2 g/L K_2_HPO_4_, 0.05 g/L MnSO_4_, 0.2 g/L MgSO_4_, 4 g/L yeast extract, and 3 g/L L-malic acid [28]. Absolute ethanol was added per formulation conditions; pH was adjusted with HCl/NaOH after constant volume. Sterile filtration (0.22 μm nylon membrane, JinTeng) was performed in a clean bench for decontamination. Three simulated wines mimicked regional conditions:

- Simulated wine A: 12% (v/v) ethanol, pH 3.60;
- Simulated wine B: 10% (v/v) ethanol, pH 3.30;
- Simulated wine C: 14% (v/v) ethanol, pH 3.80.

Activated *L. plantarum* was inoculated into wine A at 1.0% and incubated at 20 °C for 48 h. Initial (0 h) and 48 h OD_600_ were measured to calculate relative OD_600_ (48 h OD_600_ − 0 h OD_600_). The top 30 strains were selected for survival tests in simulated wine A, B, and C. Twice-activated *L. plantarum* was centrifuged (8000 r/min, 5 min), resuspended in saline, and inoculated into A, B, and C at 10^8^ CFU/mL. After 6 h incubation at 20 °C, the survival rate was calculated as (6 h viable count/0 h viable count) × 100%. Viable counts were determined via dilution plating.

### 2.3. Analysis of L-Malic Acid Content and Viable Bacterial Count

During the process, samples were collected at 24 h intervals to determine L-malic acid content and monitor the progress of MLF in simulated wine A. L-malic acid concentration was measured using a highly specific L-malic acid kit (Biosystems, Barcelona, Spain) following the manufacturer’s instructions. Measurements were conducted with the Enology Y15 automatic analyzer (Biosystems, Barcelona, Spain). Viable bacterial counts were determined by the dilution plate coating method at 24 h intervals.

### 2.4. HPLC Determination Methods for Compounds in Wine

The concentrations of glucose, fructose, glycerol, ethanol, succinic acid, citric acid, lactic acid, and tartaric acid were determined via High-Performance Liquid Chromatography (HPLC) using a Shimadzu HPLC system (Shimadzu LC-20AT, Suzhou, China). Wine samples were diluted 4-fold with ultrapure water and filtered through a 0.22 μm organic filter membrane. The HPLC conditions were as follows: C18 column (BIO-RAD 910-5025); mobile phase, 10 mmol/L H_2_SO_4_ solution; flow rate, 0.6 mL/min; column temperature, 60 °C; injection volume, 20 μL. Organic acids were detected using channel Ch3 at a wavelength of 280 nm, while sugar alcohols were detected via channel B-Ch1.

### 2.5. Analysis of Physicochemical Indices

pH, total phenol content, and total acidity were analyzed in accordance with Chinese national standard General Analytical Methods for Wine and Fruit Wine (GB/T 15038-2006) [29].

### 2.6. Analysis of Anthocyanin Contents and CIELAB Color Parameters

The concentrations of anthocyanins, including Cyanidin-3-O-glucoside (Cy 3-O-Glu), Delphinidin-3-O-glucoside (Dp 3-O-Glu), Pelargonidin-3-O-glucoside (Pt 3-O-Glu), Peonidin-3-O-glucoside (Pn 3-O-Glu), Malvidin-3-O-glucoside (Mv 3-O-Glu), Peonidin-3-O-acetylglucoside (Pn 3-O-acetylglc), Malvidin-3-O-acetylglucoside (Mv 3-O-acetylglc), Peonidin-3-O-p-coumaroylglucoside (Pn 3-O-p-coumglc trans), and Malvidin-3-O-p-coumaroylglucoside (Mv 3-O-p-coumglc trans) in wine, were analyzed using HPLC. A 1 mL wine sample was filtered using a 0.22 μm organic filter membrane. The chromatographic conditions were mobile phase A: pure H_2_O: acetonitrile: formic acid = 800:100:25. Mobile phase B: pure H_2_O: acetonitrile: formic acid = 400:500:25. The column was Agilent EC-C18, the flow rate was 1 mL/min, the column temperature was 40 °C, and the injection volume was 25 μL. The elution program was 0–4 min, 3% B; 4–14 min, 3–18% B; 14–16 min, 80% B; 16 min, 80–3% B; 16–18 min, 3% B. Anthocyanin content was quantified using dimethicalin-3-O-glucoside. The calibration curve was set to 5 concentration points, and the correlation coefficients (R^2^) were all greater than 0.999. The color intensity was quantitatively assessed using the W100 wine color analyzer (China Hanon Co., Ltd., Jinan, China) [29]. The color characteristics of the pigments were analyzed in terms of CIELAB color space parameters. Among the parameters L*, a*, b*, C*, *h_ab_*, and ΔE*_ab_, L* spans from 0 (perfect black) to 100 (perfect white) and denotes lightness; a* and b* serve as the color coordinates, where positive a* and b* values indicate red and yellow while negative values of a* and b* correspond to green and blue, respectively; C* is the chroma where C* = √[(a*)^2^ + (b*)^2^] and represents the saturation of the color: chroma values closes to 0 indicate duller or grayer colors, whereas higher values indicate more intense, vibrant colors; the hue angle (*h_ab_*), where *h_ab_* = tan^−1^(b*/a*), is a numerical value that represents the hue: hue angles of 0, 90, 180 and 270 represent red, yellow, green, and blue, respectively. *ΔE_ab_* quantifies the perceptual color difference between two colors in the CIELAB color space, calculated as *ΔE_ab_* = √ [(*ΔL*)^2^ + (Δa*)^2^ + (Δb*)^2^].

### 2.7. Analysis of Individual Phenolic Contents in Marselan Wine

The wine employed in this experiment is the 2023 vintage Marselan from Greatwall Longji Winery (Penglai, China), which has not undergone MLF treatment. The concentrations of gallic acid, catechin, vanillic acid, epicatechin, chlorogenic acid, caffeic acid, p-coumaric acid, trans-ferulic acid, quercetin, and kaempferol were determined by HPLC. To 1 mL of a wine sample, a mixture of dispersing agent (acetonitrile, 0.5 mL) and extraction agent (ethyl acetate, 1 mL) was added. After agitation for 10 s, the mixture was centrifuged at 8000 r/min for 15 min, and the supernatant was transferred to a 10 mL centrifuge tube. This procedure was repeated twice. The combined supernatants were rotary evaporated and then brought to a constant volume of 1 mL with methanol, followed by filtration through a 0.22 μm organic filter membrane. Chromatographic conditions included a Synergi Hydro-RP C18 column, with mobile phase A consisting of ultrapure water/acetonitrile/glacial acetic acid (800:100:1, v/v/v) and mobile phase B as ultrapure water/acetonitrile/glacial acetic acid (400:500:1, v/v/v); the flow rate was 1 mL/min, the injection volume was 20 μL, and the elution procedure was as follows: 0–45 min, 0–35% B; 45–50 min, 35–100% B; 50–55 min, 100% B; 55–56 min, 100–0% B; 56–62 min, 0% B. Detection wavelengths were set as follows: 280 nm for gallic acid, catechin, vanillic acid, and epicatechin; 259 nm for protocatechuic acid; 320 nm for chlorogenic acid, caffeic acid, and trans-ferulic acid; and 360 nm for quercetin and kaempferol. All pure standards are purchased from Sigma-Aldrich, with purity ≥98%. Quantification was performed using reference standards.

### 2.8. Volatile Compound Analysis

Volatile compounds were quantified using headspace solid-phase microextraction (HS-SPME) coupled with gas chromatography–mass spectrometry (GC-MS, 7890B–5975B, Agilent, Santa Clara, CA, USA). Use the PAL RSI 85 to automatically inject the sample, which can automatically select the optimal extraction head. For extraction, a 5 mL wine sample and 1 g NaCl were added to a headspace vial, followed by 10 μL of 4-methyl-2-pentanol (1 g/L) as the internal standard. The mixture was incubated at 40 °C whilst being stirred for 1 h, then subjected to SPME extraction for 30 min, and the desorption process lasted for 8 min. GC-MS conditions were as follows: helium (He) was used as the carrier gas at a constant flow rate of 1 mL/min. The oven temperature program was set as follows: initial hold at 40 °C for 3 min, ramped to 160 °C (ramp rate not specified), then increased to 230 °C at 7 °C/min, and held for 8 min. Mass spectrometry parameters included a scan range of 33–450 m/z, electron ionization (EI) in positive ion mode, and an ion source temperature of 230 °C. Unknown compounds were identified by comparing their retention times with those of standard aroma components, and quantification was performed accordingly.

### 2.9. Statistical Analysis

In this experiment, three parallel tests were performed. All data were analyzed using SPSS (version 22.0; IBM, Armonk, NY, USA) for a one-way analysis of variance (ANOVA) and Duncan’s test (*p* < 0.05). Images were drawn using Origin 2024 (OriginLab Corporation, Northampton, MA, USA) and GraphPad Prism version 8.0.2 (GraphPad Software, Boston, MA, USA).

## 3. Results and Discussion

### 3.1. Combined Stress Tolerance Screening of L. plantarum Strains

**Figure 1:**
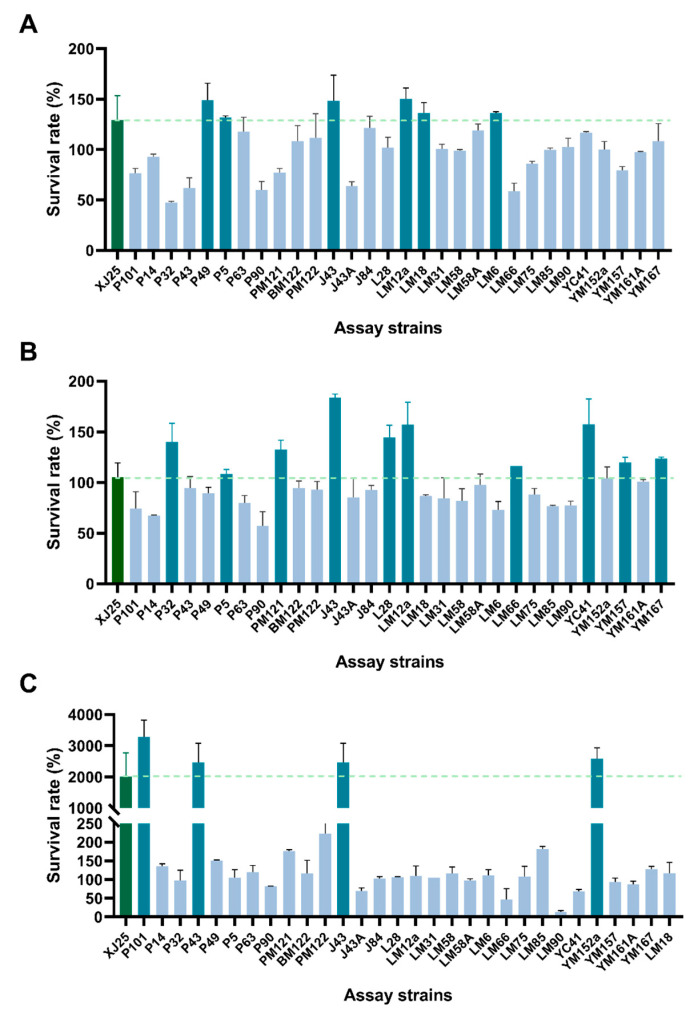
Survival rates of *L. plantarum* in three simulated wines at 6 h. (**A**) Simulated wine A condition (ethanol content was 12% and the pH was 3.60). (**B**) Simulated wine B condition (ethanol content was 10% and the pH was 3.30). (**C**) Simulated wine C condition (ethanol content was 14% and the pH was 3.80). The darker color columns indicate that this strain performs better than the control strain XJ25.

A total of 342 *L. plantarum* strains were isolated and identified from spontaneous MLF samples sourced from various winemakers across the Jiaodong Peninsula. These strains underwent initial stress screening in simulated wine A (12% ethanol, pH 3.60), a formulation mirroring the actual wine conditions of the Jiaodong Peninsula. Among the 342 isolates, 30 strains exhibited a relative OD_600_ exceeding 0.04 in simulated wine A, earning them a spot in subsequent screening rounds. In simulated wine A, six strains—P5, LM18, LM6, J43, P49, and LM12a—stood out with significantly higher 6-hour survival rates than the reference strain XJ25 (129.07%) (Figure 1A). XJ25, a well-regarded indigenous Chinese *L. plantarum*, is widely utilized in wine MLF research [30,31]. Notably, LM12a, P49, and J43 achieved survival rates of 149.92%, 148.94%, and 148.51%, respectively, surging past XJ25 and signaling robust resilience to the dual stress of 12% ethanol and pH 3.6. In stark contrast, 14 strains struggled with 6-hour survival rates below 100%, with P32 bottoming out at 47.52%. Under simulated wine B conditions (10% ethanol, pH 3.30), XJ25 maintained a 105.15% survival rate, while 10 strains—P5, LM66, YM157, YM167, PM121, P32, L28, LM12a, YC41, and J43—outperformed it (Figure 1B). As acidity rose and ethanol levels dipped, strains like LM66, YM157, YM167, PM121, P32, L28, LM12a, YC41, and J43 saw their survival rates climb, revealing a stronger aptitude for thriving in low-pH, low-ethanol environments. Among these, PM121, P32, L28, LM12a, YC41, and J43 displayed statistically significant advantages over XJ25, with J43—already a star performer in simulated wine A—leading the pack at 183.64%. Simulated wine C (14% ethanol, pH 3.80) brought another shift: XJ25 logged a 2005.63% survival rate, while five strains surpassed the 2000% mark (Figure 1C). P43, YM152a, J43, and P101 outshone XJ25 with survival rates of 2465.16%, 2575.00%, 3165.95%, and 3273.96%, respectively. Their survival rates in this high-ethanol, low-acidity milieu far outpaced those in A and B, underscoring their impressive adaptability to such conditions. Conversely, LM90 limped in with a mere 14.22% survival rate, starkly illustrating the stifling impact of high ethanol on its growth. *O. oeni* is recognized as the dominant species in MLF due to its exceptional adaptability to wine environmental stresses [5,32]. However, certain *L. plantarum* strains have also demonstrated considerable adaptive capacity [33,34], a finding supported by our screening results. Strain J43 exhibited robust adaptability across all three stress-induced simulated wine conditions, with particularly notable performance under high-acid and high-ethanol stress. In contrast, LM12a showed greater suitability for high-acid environments but was significantly impacted by elevated ethanol concentrations. Collectively, strains J43, P49, LM12a, PM121, P32, L28, P43, YM152a, and P101 displayed superior adaptability in the stress screening across the three simulated wine formulations.

### 3.2. Viable Cell Counts and L-Malic Acid Consumption During MLF with Different L. plantarum Strains

**Figure 2:**
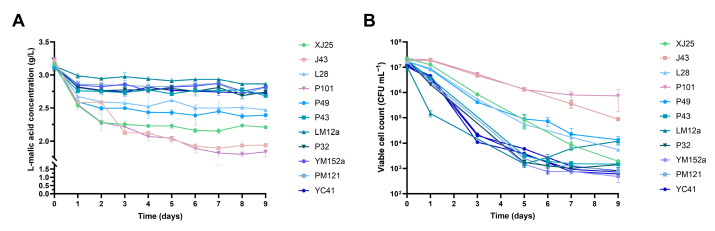
Changes in L-malic acid contents and viable cell counts of *L*. *plantarum* in simulated wine A. (**A**) Changes in malic acid contents. (**B**) Changes in viable cell counts.

Ten wild-type *L. plantarum* strains (J43, P49, LM12a, PM121, P32, L28, P43, YM152a, YC41, and P101) that exhibited superior stress tolerance in simulated wine assays were selected for MLF trials in simulated wine A (12% ethanol, pH 3.60), with strain XJ25 serving as the control. Temporal dynamics of L-malic acid concentration and viable cell counts were systematically monitored throughout fermentation. All selected strains displayed the most rapid decline in L-malic acid content within the first 24 h (Figure 2A). This phenomenon may be attributed to the highest viable cell density and biological activity observed in the simulated wine during this initial period, which drove a significant reduction in malic acid levels. The control strain XJ25 consumed the largest amount of malic acid within the first 24 h, with a decrease of 0.63 g. Strain J43 also induced a rapid reduction in malic acid content during this period. After 24 h, the rate of L-malic acid consumption by all strains slowed significantly. Among the tested strains, J43 and P101 consumed the highest amounts of L-malic acid over the entire fermentation period in wine A, ultimately reducing the malic acid concentration to below 2 g/L. Figure 2B illustrates that the viable cell counts of P101 and J43 remained relatively stable within the first 24 h, after which they began to decline. By day 9, P101 maintained the highest viable cell count, remaining above 10^6^ CFU/mL. The viable count of J43 was second only to P101, staying above 10^5^ CFU/mL—significantly higher than that of the control strain XJ25. Notably, strains P101 and J43 not only sustained high viable cell counts and robust biological activity throughout MLF but also exhibited superior L-malic acid utilization capacity compared to XJ25 during simulated wine fermentation. These findings indicate that P101 and J43 possess excellent fermentation potential. Consequently, P101 and J43 were selected for Marselan wine fermentation trials.

### 3.3. Physicochemical Indices of Wines After MLF with Different L. plantarum Strains

The basic physicochemical indices of wine samples subjected to MLF with various *L. plantarum* strains were analyzed, with details presented in Table 1. Results revealed significant alterations in several key parameters (*p* < 0.05) in wines inoculated with the screened *L. plantarum* strains for MLF compared to non-MLF controls. These parameters included pH, alcohol content, glucose, fructose, total acidity, glycerol, and total phenols. Post MLF, a notable decrease in total acidity was observed, accompanied by a significant increase in pH levels—consistent with the decarboxylation of malate to lactate during MLF [5]. All wine samples exhibited reduced fructose content after fermentation, indicating fructose metabolism by these strains. Glycerol concentrations also differed significantly post fermentation, suggesting glycerol metabolism was caused by the three strains; J43 showed the lowest glycerol concentration at 4.34 g/L, representing a 0.51 g reduction. Glycerol undergoes aerobic metabolism via the glycerol kinase pathway, where it is phosphorylated to glycerol-3-phosphate and subsequently oxidized to dihydroxyacetone phosphate. Notably, 13 glycerol-metabolizing *L. plantarum* strains have been isolated from Australian wines [35]. Alcohol content decreased significantly in J43-inoculated wines, reaching 13.68% (v/v), while only slight reductions were observed in XJ25 and P101 samples. Additionally, total phenol content decreased post MLF in all inoculated wines, with J43-inoculated samples showing the lowest total phenol content (3047.19 mg/L)—a value significantly different from pre-fermentation levels.

### 3.4. Changes in Viable L. plantarum Counts and Organic Acid Contents in Marselan Wine

**Figure 3:**
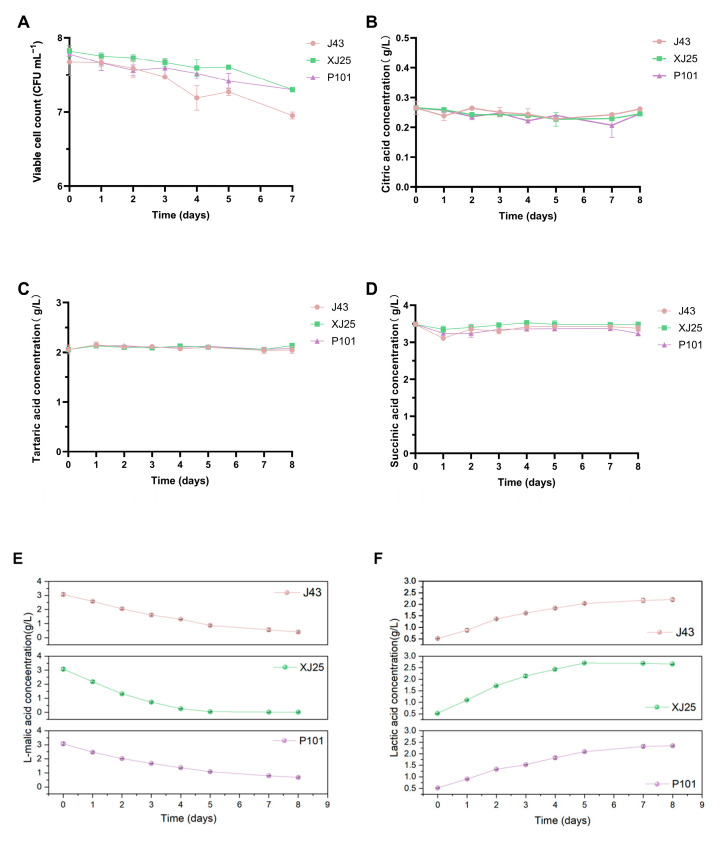
Dynamics of organic acid contents and viable cell counts during MLF of wine with different strains. (**A**) Viable cell counts. (**B**) Citric acid concentrations. (**C**) Tartaric acid concentrations. (**D**) Succinic acid concentrations. (**E**) L-malic acid concentrations. (**F**) Lactic acid concentrations.

During MLF, the viable cell counts of the three strains remained consistently above 10^7^ CFU/mL, with only a slight reduction, indicating good adaptability and viability in Marselan wine (Figure 3A). Acid stress significantly impacts industrial microbial processes [36], particularly in food fermentation, where it inhibits microbial growth, disrupts metabolic activity, and prolongs fermentation duration [37]. As shown in Figure 3B–D, the contents of citric acid, tartaric acid, and succinic acid in wines inoculated with XJ25, P101, and J43 remained essentially unchanged throughout MLF. Citric acid can serve as a carbohydrate source to provide energy and accelerate the growth of lactic acid bacteria; studies have shown that citrate supplementation can increase the abundance of *Micrococcus* and *Lactobacillus* [38]. However, in this experiment, the three *L. plantarum* strains did not metabolize citric acid, resulting in no significant change in citric acid content in the wine.

The initial concentration of L-malic acid in the wine was 3.07 g/L, with an initial lactic acid content of 0.53 g/L. P101 and J43 exhibited a rapid rate of L-malic acid consumption in the first 5 days, which slowed thereafter. By day 8, the L-malic acid concentrations were 0.41 g/L for P101 and 0.68 g/L for J43 (Figure 3E). The lactic acid concentrations reached 2.21 g/L for J43 and 2.35 g/L for P101 (Figure 3F), with no significant difference in the extent of lactate accumulation between the two strains. XJ25 showed the fastest malate consumption rate: by day 5, L-malic acid in the wine was completely depleted, and the lactate concentration remained essentially unchanged, marking the end of MLF. While J43 and P101 consumed L-malic acid at a slower pace than XJ25, they maintained robust viable cell counts throughout fermentation, achieved substantial lactate production, and preserved key organic acid profiles—collectively reflecting their excellent fermentation potential for winemaking.

### 3.5. Effects of L. plantarum on Wine Color and Anthocyanin Contents Before and After MLF

Table 2 reveals that MLF induced by *L. plantarum* in Marselan wine led to an increase in wine brightness, as reflected by a rise in L* value from the initial 16.86. Among the strains tested, P101 yielded the highest L* value (19.20), indicating significantly greater brightness and enhanced gloss. However, no significant differences were observed in other color parameters across treatments, including a*, b*, C*_ab_ (Chroma), and h*_ab_ (Hue angle). The total color differences (ΔE*_ab_) between wines before and after MLF were below 3.0 for all strains, suggesting visually indiscernible color alterations. Collectively, these results indicate that MLF mediated by these strains exerts minimal impact on overall wine color.

**Figure 4:**
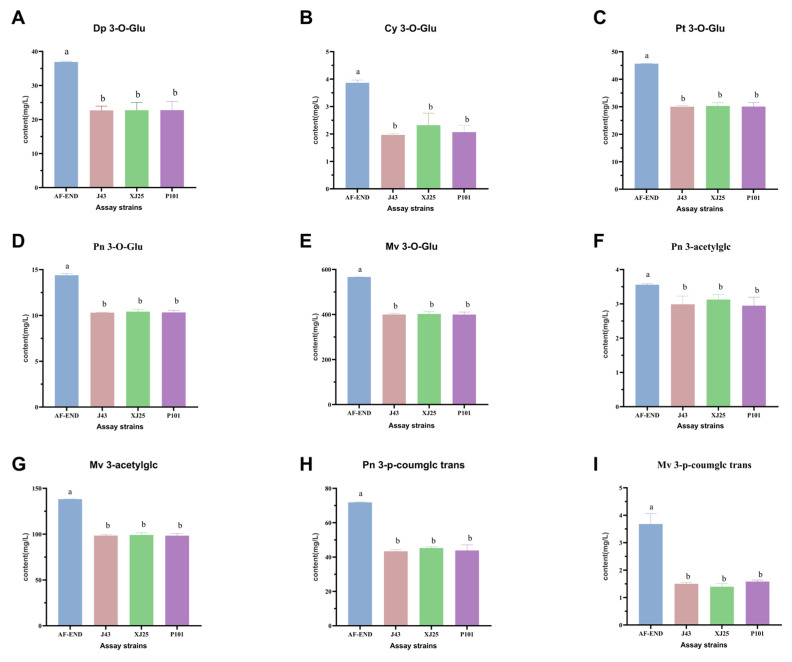
Anthocyanin contents before and after MLF. (**A**) Dp 3-O-Glu. (**B**) Cy 3-O-Glu. (**C**) Pt 3-O-Glu. (**D**) Pn 3-O-Glu. (**E**) Mv 3-O-Glu. (**F**) Pn 3-acetylglc. (**G**) Mv 3-acetylglc. (**H**) Pn 3-p-coumglc trans. (**I**) Mv 3-p-coumglc trans. AF-END indicates the wine before MLF. Different lowercase letters in each of these columns indicate a significant difference (*p* < 0.05), and the same letter indicates that the difference is not significant (*p* > 0.05).

Figure 4 illustrates that before MLF, malvidin-3-O-glucoside (Mv 3-O-Glu) was the most abundant anthocyanin in the wine at 566.54 mg/L, while peonidin-3-O-acetylglucoside (Pn 3-O-acetylglc) was the least abundant at 3.56 mg/L. After MLF, the content of all tested anthocyanins in wine samples had decreased, with no significant differences observed between fermentations using different strains. Compared to wines fermented with XJ25, those fermented with J43 and P101 showed higher levels of trans-malvidin-3-p-coumaroylglucoside (Mv 3-p-coumglc trans) and lower levels of cyanidin-3-O-glucoside (Cy 3-O-Glu), while the contents of the other seven anthocyanins were nearly identical. The detected anthocyanins content following MLF was lower than that observed prior to MLF. Critically, despite reduced monomeric anthocyanin content after MLF, combined with the observed color stability (ΔE*_ab_ < 3.0; Table 2), this phenomenon likely stems from *L. plantarum*’s ability to release acetaldehyde—a key mediator promoting anthocyanin polymerization and enhancing color stability [39].

**Figure 5:**
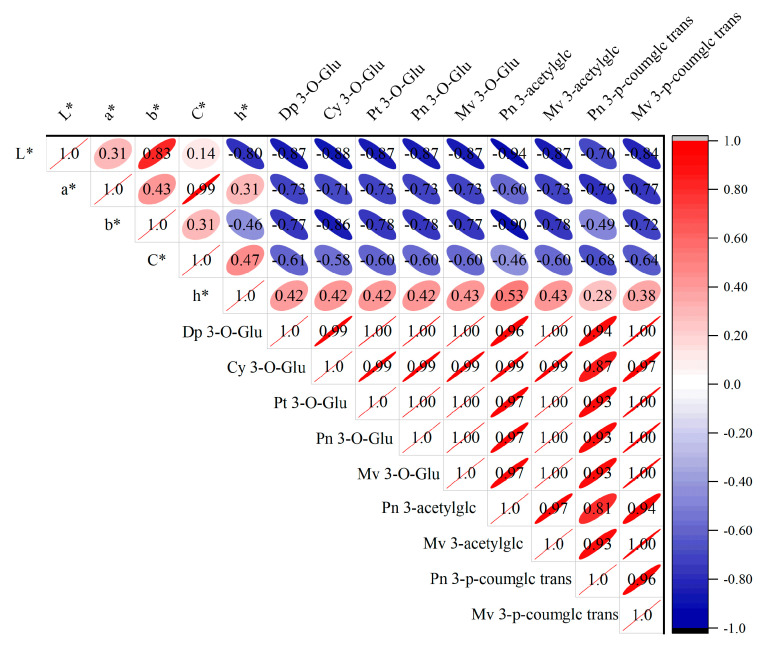
Heatmap of correlation between anthocyanins and CIELAB parameters.

Pearson correlation analysis was performed to explore intrinsic relationships between wine anthocyanin content and color parameters (Figure 5), whose core purpose is to reveal the intrinsic relationship between the content changes of nine monomeric anthocyanins and five CIELAB color parameters (lightness L*, redness a*, yellowness b*, chroma *C_ab_*, hue angle h_ab_*) in Marselan wine after malolactic fermentation (MLF) with different *L. plantarum* strains. Results showed positive correlations among L*, a*, b*, and C*_ab_: higher brightness (L*) was associated with a more intense red hue (a*), more prominent yellow hue (b*), and greater saturation (C*_ab_). The correlation between a and C*_ab_ was particularly strong (correlation coefficient = 0.99), indicating a high degree of linearity. All nine anthocyanins exhibited positive correlations with each other, with correlation values exceeding 0.8, reflecting an extremely strong linear relationship. In contrast, L*, a*, b*, and C*_ab_ were negatively correlated with the nine anthocyanins, suggesting that higher anthocyanin diversity and content were associated with lower wine brightness, less intense red and yellow hues, and reduced saturation. The hue angle (h*_ab_) showed negative correlations with L* and b*, but positive correlations with a* and C*_ab_. These results indicate that the reduction in anthocyanin content post MLF contributed to brighter, more saturated, and more transparent wines, exerting a positive impact on overall wine color. Notable differences were observed in the correlations between anthocyanin profiles (types and contents) and color parameters when comparing wines undergoing MLF with *O. oeni* versus those fermented with *L. plantarum* [30].

### 3.6. Individual Phenolic Contents During MLF with Different L. plantarum Strains

**Figure 6:**
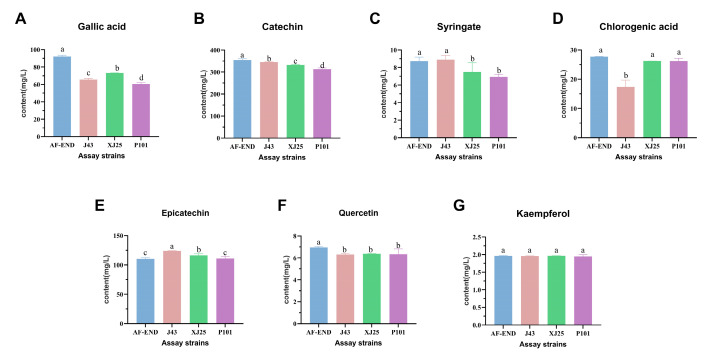
Individual phenolics before and after MLF. (**A**) Gallic acid. (**B**) Catechin. (**C**) Syringate. (**D**) Chlorogenic acid. (**E**) Epicatechin. (**F**) Quercetin. (**G**) Kaempferol. AF-END indicates the wine before MLF. Different lowercase letters in each of these columns indicate a significant difference (*p* < 0.05), and the same letter indicates that the difference is not significant (*p* > 0.05).

Phenolic compounds are critical components of wine, influencing its color [40], taste, and structural properties, while also being associated with antioxidant activity [41] and potential health benefits [42]. Seven individual phenolics were detected via HPLC (Figure 6). Among these, kaempferol was the least abundant at 1.96 mg/L, whereas catechin was the most abundant at 354.92 mg/L. Gallic acid content decreased significantly post MLF, with strain-specific variations: P101 induced the greatest reduction, with content dropping from an initial 90.02 mg/L to 60.55 mg/L. MLF mediated by the tested *L. plantarum* strains also reduced catechin content, with P101 causing the most substantial decline—from 354.9 mg/L to 313.6 mg/L. Syringic acid content remained relatively unchanged in J43-inoculated wines post MLF but decreased in those fermented with XJ25 and P101. In contrast, chlorogenic acid content decreased specifically in wines fermented with J43.

### 3.7. Volatile Compound and PCA of Marselan Wines Fermented with Different L. plantarum Strains

**Figure 7:**
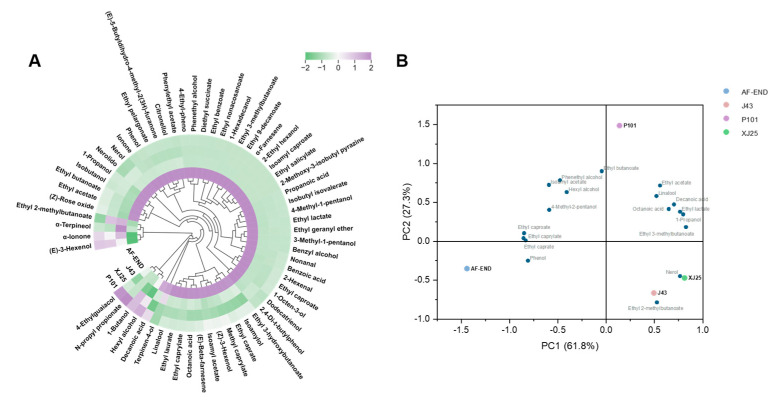
Aroma components analysis of superior indigenous strains in Marselan wines (**A**) Heatmap of aroma substances produced by different strains in Marselan wine. (**B**) PCA plot of volatile compounds in Marselan wine after fermentation with different strains. AF-END indicates the wine before MLF.

*L. plantarum* strains exhibit a more diverse enzyme profile compared to *O. oeni* strains, particularly in terms of aroma-modifying enzymes such as β-glucosidase and phenolic acid decarboxylase [34]. Qualitative and quantitative analyses of volatile components in Marselan wine were conducted post AF and post MLF with three *L. plantarum* strains. As shown in Table 3, a total of 62 aroma compounds were detected in Marselan wine after AF, comprising 20 esters, 13 alcohols, 14 terpenes, 5 Aldehydes and Ketones, 4 volatile phenols, 5 fatty acids, and 1 pyrazine. Alcohols accounted for the highest total content among all volatile compounds in the wine samples, with four substances exhibiting an odor activity value (OAV) exceeding 0.1. Post MLF, the total alcohol content decreased: P101-fermented wines showed the highest total alcohol concentration (123,826.78 μg/L), while XJ25-fermented wines had the lowest (93,050.44 μg/L). Esters represented the most diverse class of volatile compounds, contributing fruity notes to the wine aroma. Among these, five substances had an OAV greater than 1. After MLF, the contents of ethyl acetate increased in all strains, with P101 showing significantly higher levels than XJ25. Notably, J43 fermentation resulted in a three-fold increase in ethyl 2-methylbutyrate compared to pre-MLF levels, which is known to make a significant contribution to the fruity aroma characteristics of wine [43,44]. As illustrated in the heatmap (Figure 7A), MLF with *L. plantarum* strains increased ethyl lactate content, which may impart richer milk and butter notes, thereby enhancing the complexity and elegance of the wine aroma [45]. Additionally, MLF with *L. plantarum* strains led to increased contents of isobutanol, 1-heptanol, and benzyl alcohol in Marselan wine, which can contribute orange-like and fatty aromas, while other alcohols remained relatively unchanged.

Terpenes, as signature components of grape varieties, contribute floral, citrus, and tropical fruit aromas [46]. MLF with J43 and P101 strains increased the contents of linalool and nerol in wine samples, which are associated with floral and citrus aromas. MLF with all three *L. plantarum* strains increased the contents of fatty acids such as octanoic acid; at low concentrations, these can impart lactic acid notes, but at high concentrations, they may introduce fatty or other undesirable odors [47]. No significant differences in aldehyde and ketone contents were observed in wine samples before and after MLF.

To further characterize the differences in volatile compounds among wines fermented with different strains, principal component analysis (PCA) was performed on substances with OAVs exceeding 0.1 across all treatment groups. As shown in Figure 7B, the first principal component (PC1) accounted for 61.8% of the total variance, while the second principal component (PC2) explained 27.2%. Most esters, alcohols, and terpenes were concentrated in the first and second quadrants, with ethyl 2-methylbutyrate and nerol localized in the fourth quadrant and phenolic compounds in the third quadrant. Non-MLF wine samples clustered in the third quadrant, whereas wines fermented with J43 and XJ25 were positioned in the fourth quadrant, closely associated with ethyl 2-methylbutyrate and nerol—compounds known to contribute apple-like aroma and floral/grass characteristics to Marselan wine. Substances with positive contributions to wine aroma, including ethyl acetate, linalool, ethyl lactate, and ethyl isovalerate, were clustered in the first quadrant. Notably, P101-fermented wines also fell within this quadrant, exhibiting a proximity to ethyl butanoate, ethyl acetate, and linalool, compounds associated with characteristic fruity aromas such as banana, strawberry, and pineapple. The clear separation observed in the PCA plot between Marselan wines subjected to *L. plantarum*-mediated MLF and non-MLF samples indicates that MLF with *L. plantarum* strains enhances the aroma complexity of Marselan wine.

## 4. Conclusions

In this study, 342 indigenous *L. plantarum* strains were characterized, among which 30 strains exhibited robust tolerance to harsh winemaking environments. Through comprehensive evaluation, the strains J43 and P101 were identified as superior, showing excellent malic acid consumption capacity and adaptability to winemaking stress factors. During MLF, these strains efficiently consumed L-malic acid and produced lactic acid within 8 days. MLF mediated by J43 and P101 significantly influenced anthocyanin contents in wines, while altering organic acid contents. However, these strains had almost no effect on wine basic physicochemical parameters, color parameters, and individual phenolic contents. Comprehensive aroma profiling of Marselan wines undergoing MLF revealed that J43 and P101 enhanced the production of some key aroma compounds and increased contents of ethyl acetate, ethyl lactate, ethyl 2-methylbutyrate, and nerol, as well as other such compounds, contributing to more intense aromatic characteristics. The selection of these indigenous strains offers distinctive wine attributes while supporting oenological biodiversity. As *L. plantarum* has not been widely adopted as wine starter culture, the strains J43 and P101 present significant potential for application in wine production and as innovative fermentation agents, thereby creating new avenues for enhancing winemaking quality and diversity. This study also highlights the importance of regional microbial biodiversity in developing locally adapted winemaking solutions, which contribute to innovation and sustainability in the global wine industry.

## Figures and Tables

**Table 1:** Physicochemical indices of wines before and after MLF.

Strain	pH	Total Acid g/L	Glucose g/L	Fructose g/L	Glycerin g/L	Alcohol (%) (v/v)	Total Phenol mg/L
AF-END	3.61 ± 0.00 ^c^	7.04 ± 0.15 ^a^	1.39 ± 0.01 ^c^	7.72 ± 0.10 ^a^	4.85 ± 0.14 ^a^	14.26 ± 0.08 ^a^	3504.96 ± 186.20 ^a^
J43	3.76 ± 0.00 ^b^	5.90 ± 0.17 ^b^	1.37 ± 0.03 ^c^	6.24 ± 0.17 ^b^	4.34 ± 0.10 ^b^	13.68 ± 0.38 ^b^	3047.19 ± 74.38 ^b^
XJ25	3.79 ± 0.00 ^a^	5.30 ± 0.34 ^c^	1.54 ± 0.02 ^a^	5.93 ± 0.06 ^b^	4.42 ± 0.03 ^b^	14.02 ± 0.06 ^ab^	3411.33 ± 57.70 ^a^
P101	3.79 ± 0.00 ^a^	5.06 ± 0.29 ^c^	1.46 ± 0.04 ^b^	6.06 ± 0.30 ^b^	4.41 ± 0.02 ^b^	13.99 ± 0.10 ^ab^	3264.50 ± 168.12 ^ab^

The mean ± SD of measurements made in triplicates was used to reflect the characteristic values. Different lowercase letters in each of these columns indicate a significant difference (*p* < 0.05), and the same letter indicates that the difference is not significant (*p* > 0.05). AF-END indicates the wine before MLF.

**Table 2:** CIELAB color parameters of wines before and after MLF.

Strain	L*	a*	b*	C*_ab_	h*_ab_	*△* E*_ab_
AF-END	16.86 ± 0.21 ^c^	45.25 ± 0.35 ^a^	20.53 ± 0.26 ^a^	49.68 ± 0.42 ^a^	0.43 ± 0.01 ^a^	-
J43	18.26 ± 0.67 ^bc^	46.54 ± 1.03 ^a^	21.58 ± 1.32 ^a^	51.04 ± 1.25 ^a^	0.43 ± 0.03 ^a^	2.17 ± 0.25 ^b^
XJ25	18.13 ± 0.62 ^bc^	46.41 ± 0.86 ^a^	20.85 ± 0.63 ^a^	50.88 ± 1.04 ^a^	0.42 ± 0.01 ^a^	1.75 ± 0.22 ^c^
P101	19.20 ± 1.22 ^a^	45.57 ± 3.51 ^a^	21.50 ± 0.60 ^a^	49.75 ± 4.12 ^a^	0.44 ± 0.03 ^a^	2.55 ± 0.40 ^a^

The mean ± SD of measurements made in triplicates was used to reflect the characteristic values. Different lowercase letters in each of these columns indicate a significant difference (*p* < 0.05), and the same letter indicates that the difference is not significant (*p* > 0.05). AF-END indicates the wine before MLF.

**Table 3:** Concentrations, odor thresholds, and aroma descriptors of volatile compounds in the wines before and after MLF with different strains.

Compounds	Aroma Concentration (μg/L)				Thresholds	OAV	Description
	AF-END	J43	XJ25	P101			
Esters (20)							
Ethyl acetate	31,039.50 ± 187.59 ^d^	33,513.88 ± 331.35 ^c^	37,143.24 ± 107.61 ^b^	39,475.58 ± 2466.94 ^a^	7500	>1	Banana, Strawberry
Ethyl butanoate	110.39 ± 1.74 ^b^	101.86 ± 1.36 ^c^	111.98 ± 2.62 ^b^	122.11 ± 5.25 ^a^	400	>0.1	Strawberry, Banana, Pineapple
Ethyl 2-methylbutanoate	1.44 ± 0.33 ^b^	6.71 ± 0.07 ^a^	6.02 ± 0.91 ^a^	0.00 ± 0.00 ^c^		>1	Apple
N-propyl propionate	0.00 ± 0.00 ^b^	0.00 ± 0.00 ^b^	0.00 ± 0.00 ^b^	6.69 ± 0.60 ^a^			
Ethyl 3-methylbutanoate	0.70 ± 0.02 ^b^	0.94 ± 0.02 ^a^	0.93 ± 0.03 ^a^	0.92 ± 0.06 ^a^	3	>0.1	Strawberry, Sweet Fruity
Isoamyl acetate	140.24 ± 4.96 ^a^	131.82 ± 2.54 ^a^	130.62 ± 7.22 ^a^	141.67 ± 0.60 ^a^	160	>0.1	Banana, Fruity
Isobutyl isovalerate	86.09 ± 1.00 ^a^	85.31 ± 0.04 ^a^	87.45 ± 4.63 ^a^	88.51 ± 3.63 ^a^			
Ethyl caproate	183.10 ± 1.95 ^a^	126.95 ± 1.86 ^c^	124.48 ± 1.25 ^c^	145.04 ± 5.67 ^b^	14	>1	Green apple, Strawberry
Ethyl lactate	5696.61 ± 109.69 ^c^	8452.46 ± 72.65 ^b^	10,514.85 ± 890.43 ^a^	10,125.87 ± 617.30 ^a^	14,000	>0.1	Milk, Butter
Methyl caprylate	41.82 ± 1.17 ^a^	0.21 ± 0.04 ^b^	0.01 ± 0.00 ^b^	0.44 ± 0.06 ^b^			
Ethyl caprylate	806.04 ± 4.23 ^a^	483.09 ± 10.60 ^c^	408.13 ± 17.75 ^d^	549.00 ± 18.75 ^b^	5	>1	Pineapple, Pear, Floral
Ethyl 3-hydroxybutanoate	259.54 ± 0.03 ^b^	282.64 ± 3.83 ^a^	278.41 ± 11.78 ^ab^	294.31 ± 14.40 ^a^	200,000	<0.1	
Ethyl pelargonate	12.95 ± 0.15 ^a^	9.89 ± 0.24 ^b^	7.31 ± 0.62 ^c^	9.11 ± 0.37 ^b^	200	<0.1	Fruity
Ethyl caprate	551.25 ± 10.49 ^a^	417.11 ± 4.64 ^b^	339.74 ± 17.62 ^c^	426.03 ± 8.34 ^b^	200	>1	Fruity
Diethyl succinate	101.82 ± 1.08 ^b^	112.89 ± 3.12 ^a^	105.02 ± 0.91 ^b^	111.31 ± 0.06 ^a^	6000	<0.1	Fruity, Melon
Ethyl benzoate	3.99 ± 0.00 ^a^	3.98 ± 0.01 ^a^	3.95 ± 0.01 ^b^	3.96 ± 0.00 ^b^			
Ethyl 9-decanoate	0.00 ± 0.00 ^c^	1.09 ± 0.12 ^a^	0.18 ± 0.00 ^bc^	0.32 ± 0.03 ^b^			
Phenylethyl acetate	12.41 ± 0.16 ^a^	12.21 ± 0.39 ^ab^	10.97 ± 0.34 ^c^	11.41 ± 0.13 ^bc^	250	<0.1	Rose, Sweet
Ethyl laurate	9.11 ± 0.00 ^ab^	9.32 ± 0.35 ^a^	8.43 ± 0.31 ^b^	9.37 ± 0.27 ^a^	1500	<0.1	Sweet, Beeswax
Ethyl nonacosanoate	5.53 ± 0.02 ^a^	5.66 ± 0.07 ^a^	5.64 ± 0.11 ^a^	5.65 ± 0.06 ^a^			
Alcohols (13)							
1-Propanol	24,180.88 ± 210.70 ^ab^	21,199.51 ± 567.61 ^b^	26,965.99 ± 2592.89 ^a^	26,482.49 ± 1510.11 ^a^	306,000	<0.1	Mello, Mature fruity, Floral and Green
Isobutanol	20,490.93 ± 572.41 ^b^	21,429.31 ± 98.03 ^a^	21,817.10 ± 117.27 ^a^	21,755.57 ± 438.80 ^a^	40,000	>0.1	Chemical
1-Butanol	1008.94 ± 5.06 ^b^	1007.35 ± 0.65 ^b^	1043.25 ± 15.06 ^a^	1043.41 ± 3.64 ^a^	150,000	<0.1	Fruity, Green, Malt, Chemical, Alcohol
4-Methyl-2-pentanol	2066.00 ± 0.00 ^a^	2066.00 ± 0.00 ^a^	2066.00 ± 0.00 ^a^	2066.00 ± 0.00 ^a^			
Isoamylol	59,476.92 ± 258.64 ^a^	50,966.47 ± 129.62 ^c^	26,789.52 ± 816.49 ^d^	57,480.29 ± 1242.33 ^b^	30,000	>1	Caramel, Lipid
4-Methyl-1-pentanol	20.08 ± 0.04 ^ab^	18.84 ± 0.31 ^c^	19.93 ± 0.16 ^b^	20.47 ± 0.09 ^a^	50,000	<0.1	
3-Methyl-1-pentanol	16.08 ± 0.13 ^a^	13.59 ± 0.24 ^b^	15.62 ± 0.23 ^a^	16.43 ± 0.75 ^a^	500	<0.1	
Hexyl alcohol	837.77 ± 1.03 ^b^	766.70 ± 8.22 ^c^	817.96 ± 7.20 ^b^	844.34 ± 14.77 ^a^	8000	<0.1	
1-Heptanol	28.59 ± 0.08 ^a^	27.23 ± 0.15 ^b^	27.49 ± 0.03 ^b^	27.74 ± 0.22 ^b^	2500	<0.1	
Octanol	5.21 ± 0.20 ^a^	3.14 ± 0.01 ^c^	2.84 ± 0.04 ^c^	3.50 ± 0.21 ^b^	40	>0.1	Floral
1-Decanol	28.41 ± 2.12 ^c^	52.91 ± 1.00 ^a^	46.23 ± 1.94 ^b^	56.45 ± 2.10 ^a^	400	>0.1	Orange, Fatty
Benzyl alcohol	537.12 ± 2.66 ^c^	589.82 ± 11.65 ^b^	630.14 ± 23.13 ^a^	618.40 ± 20.07 ^ab^	200,000	<0.1	Roast, Fruity
Phenethyl alcohol	12,456.63 ± 35.15 ^a^	12,143.24 ± 1000.30 ^a^	11,895.77 ± 395.40 ^a^	12,682.42 ± 867.55 ^a^	400	>1	Orange, Fatty
Terpenes (14)							
Limonene	0.88 ± 0.07 ^a^	0.30 ± 0.00 ^b^	0.58 ± 0.04 ^ab^	0.77 ± 0.16 ^ab^	10	>0.1	Sweet, Citrus, lemon
Linalool	21.66 ± 0.01 ^c^	21.83 ± 0.03 ^a^	21.74 ± 0.01 ^b^	21.87 ± 0.03 ^a^	25	>0.1	Floral, Citrus
1-Octen-3-ol	15.11 ± 0.10 ^a^	13.56 ± 0.11 ^c^	13.30 ± 0.23 ^c^	14.12 ± 0.34 ^b^			
(E)-3-Hexenol	38.01 ± 1.45 ^a^	33.42 ± 0.03 ^b^	35.02 ± 0.37 ^b^	35.19 ± 0.65 ^b^			
(Z)-Rose oxide	3.35 ± 0.00 ^a^	3.34 ± 0.00 ^c^	3.35 ± 0.00 ^d^	3.35 ± 0.00 ^b^			
(Z)-3-Hexenol	1.64 ± 0.02 ^d^	20.21 ± 0.10 ^b^	17.85 ± 0.56 ^c^	24.47 ± 1.86 ^a^			
α-Terpineol	12.36 ± 0.12 ^b^	19.96 ± 6.09 ^a^	12.13 ± 0.03 ^b^	12.70 ± 0.11 ^b^	250	<0.1	Lilac
Nerol	127.68 ± 1.17 ^c^	145.60 ± 10.33 ^ab^	148.60 ± 2.52 ^a^	133.38 ± 0.52 ^b^	400	>0.1	Floral, Green
Ethyl geranyl ether	12.12 ± 0.02 ^a^	12.02 ± 0.03 ^a^	12.09 ± 0.10 ^a^	12.16 ± 0.11 ^a^			
Terpinen-4-ol	9.88 ± 0.02 ^b^	12.39 ± 1.68 ^a^	10.38 ± 0.05 ^ab^	10.38 ± 0.03 ^ab^			
(E)-Beta-farnesene	3219.34 ± 38.03 ^a^	2256.72 ± 40.30 ^c^	1935.72 ± 64.89 ^d^	2417.84 ± 61.92 ^b^			
Citronellol	8.40 ± 0.35 ^a^	6.87 ± 0.69 ^b^	5.95 ± 0.16 ^b^	6.50 ± 0.09 ^b^	100	<0.1	Green, Lilac, Rose
Nerolido	9.83 ± 0.02 ^a^	9.95 ± 0.21 ^a^	9.97 ± 0.27 ^a^	9.83 ± 0.02 ^a^	400	<0.1	Green, Floral
Dodecatrienol	81.28 ± 0.00 ^b^	83.38 ± 1.62 ^ab^	85.22 ± 0.95 ^a^	82.61 ± 0.35 ^b^			
Aldehydes and Ketones (5)							
2-Hexenal	401.26 ± 1.77 ^a^	244.66 ± 11.87 ^c^	237.31 ± 2.94 ^c^	291.43 ± 16.09 ^b^			
Nonanal	0.02 ± 0.01 ^a^	0.00 ± 0.00 ^b^	0.00 ± 0.00 ^b^	0.01 ± 0.01 ^b^	2.5	<0.1	Citrus
α-Ionone	10.54 ± 0.25 ^a^	9.96 ± 0.19 ^ab^	8.74 ± 0.10 ^c^	9.40 ± 0.67 ^bc^			
(E)-5-Butyldihydro-4-methyl-2(3H)-furanone	227.48 ± 0.01 ^c^	227.84 ± 0.15 ^a^	227.46 ± 0.12 ^c^	227.69 ± 0.09 ^ab^			
Ionone	9.45 ± 0.01 ^a^	9.47 ± 0.01 ^a^	9.45 ± 0.01 ^a^	9.45 ± 0.00 ^a^			
Volatile Phenols (4)							
Phenol	16.16 ± 0.01 ^a^	15.48 ± 0.73 ^ab^	15.07 ± 0.10 ^b^	15.27 ± 0.01 ^b^	30	>0.1	
4-Ethylguaiacol	125.70 ± 0.03 ^b^	125.56 ± 0.02 ^c^	125.62 ± 0.03 ^c^	125.79 ± 0.04 ^a^			
4-Ethyl-pheno	66.10 ± 0.02 ^a^	66.16 ± 0.09 ^a^	66.12 ± 0.04 ^a^	66.21 ± 0.03 ^a^			
2,4-Di-t-butylphenol	177.36 ± 0.10 ^a^	179.34 ± 5.71 ^a^	185.39 ± 8.94 ^a^	181.06 ± 2.40 ^a^			
Fatty acids (5)							
Propanoic acid	8232.04 ± 120.08 ^b^	8853.37 ± 137.58 ^a^	8869.48 ± 261.78 ^a^	8880.14 ± 243.06 ^a^			
Isovaleric acid	80.37 ± 65.62 ^c^	196.47 ± 16.12 ^b^	302.09 ± 15.53 ^a^	202.13 ± 16.33 ^b^	3000	<0.1	Sour, Cheese
Octanoic acid	353.73 ± 7.14 ^b^	498.00 ± 65.80 ^a^	435.19 ± 14.94 ^ab^	501.68 ± 30.48 ^a^	500	>1	Sour, Cheese, Fatty
Decanoic acid	188.28 ± 2.28 ^c^	245.13 ± 17.88 ^ab^	232.35 ± 5.00 ^b^	255.88 ± 5.69 ^a^	1000	>0.1	Sour, Fatty
Benzoic acid	23,418.69 ± 3.04 ^a^	23,416.63 ± 5.84 ^a^	23,423.12 ± 9.65 ^a^	23,433.27 ± 9.87 ^a^			
Other (1)							
2-Methoxy-3-isobutyl pyrazine	0.94 ± 0.00 ^a^	0.93 ± 0.00 ^b^	0.93 ± 0.00 ^b^	0.93 ± 0.00 ^b^			

The mean ± SD of measurements made in triplicates were used to reflect the characteristic values. Different lowercase letters in each of these columns indicate a significant difference (*p* < 0.05), and the same letter indicates that the difference is not significant (*p* > 0.05). AF-END indicates the wine before MLF.

## Data Availability

The original contributions presented in this study are included in the article. Further inquiries can be directed to the corresponding authors.
